# Effects of Boron Carbide on Coking Behavior and Chemical Structure of High Volatile Coking Coal during Carbonization

**DOI:** 10.3390/ma14020302

**Published:** 2021-01-08

**Authors:** Qiang Wu, Can Sun, Zi-Zong Zhu, Ying-Dong Wang, Chong-Yuan Zhang

**Affiliations:** College of Materials Science and Engineering, Chongqing University, Chongqing 400044, China; qiangwucqu@163.com (Q.W.); suncan2230@163.com (C.S.); 18243934868@163.com (Y.-D.W.); zhangchongyuan0811@163.com (C.-Y.Z.)

**Keywords:** high volatile coking coal, boron carbide, coking behavior, chemical structure, coke quality

## Abstract

Modified cokes with improved resistance to CO_2_ reaction were produced from a high volatile coking coal (HVC) and different concentrations of boron carbide (B_4_C) in a laboratory scale coking furnace. This paper focuses on modification mechanism about the influence of B_4_C on coking behavior and chemical structure during HVC carbonization. The former was studied by using a thermo-gravimetric analyzer. For the latter, four semi-cokes prepared from carbonization tests for HVC with or without B_4_C at 450 °C and 750 °C, respectively, were analyzed by using Fourier transform infrared spectrum and high-resolution transmission electron microscopy technologies. It was found that B_4_C will retard extensive condensation and crosslinking reactions by reducing the amount of active oxygen obtained from thermally produced free radicals and increase secondary cracking reactions, resulting in increasing size of aromatic layer and anisotropic degree in coke structure, which eventually improves the coke quality.

## 1. Introduction

Adding cheap materials into coal blends to produce metallurgical coke has been extensively researched, due to the gradual rise of coking coal price and inadequate supply of the prime-coking coals with medium volatility. The most studied method is harnessing non-coking coal to replace part of coking coals, but the caking property of coal blends will deteriorate in such a situation. To improve the caking property, on the one hand, various high bond-ability substances, such as pitches, coal tar pitches, coal extracts, and solvent-refined coals, have been used in the carbonization process of coal blending with low bond-ability [1,2,3,4,5,6,7]. On the other hand, non-coking coal was pretreated by thermal treatments [8], hydrothermal treatments [9,10], and steam treatments [11,12]. Additionally, to reduce the costs of coal blends and the amount of CO_2_ emission, adding small amount of biomass material into coal blends to produce metallurgical coke has also been suggested [13,14,15]. Although these methods can broaden coking coal resources, few industrial applications about the above studies are successful because of factors such as coke quality, cost, production conditions, etc.

It is widely known that the reserve of high volatile coking coal (HVC) occupies about half of all coking coal reserves, and the price of HVC is relatively cheap. Therefore, increasing the usage amount of HVC in coal blending to produce metallurgical coke is also an effective solution to reduce the cost of coke and maintain the sustainable development of the coke industry. However, the usage amount of HVC in coal blending is usually limited to 20–30% in the producing process of metallurgical coke [5,12], which is extremely disproportionate to the reserve of HVC. This is because HVC is a low proportion of metamorphism coking coal and possesses plenty of oxygen-containing groups as well as aliphatic side chains, which results in the rapid formation of large quantities of gases, free radicals, and plastic mass with high fluidity during HVC carbonization, subsequently leaving a weak coke with a thin-walled porous structure [13,14,16,17,18,19]. In such a case, Qian et al found that the addition of pitches could contribute to developing intermediate texture during HVC carbonization [20]. This improves the coke quality, but the complex operation process and plugging problem limit its industrial application. Vega et.al., asserted that mild oxidation was an effective method to improve the coking performance of HVC with low oxygen content (less than 5%) [19]. However, HVC usually contains high oxygen content (more than 10%) so that mild oxidation will lead to excessive oxygen in HVC, which worsens its thermoplasticity and coking performance.

Admittedly, exploring a new method which is able to improve the coking performance of HVC, finally increasing its usage in coal blends, is urgent and essential. Previous studies have indicated that during resin pyrolysis, B_4_C reacts with oxygen-containing fragments released from resin to generate boric oxide, whose formation further affects the viscosity of resin at high temperature [21,22]. The compositions of the oxygen-containing fragments obtained from the pyrolysis process of resin and HVC are partly similar; that B_4_C may react with the oxygen-containing fragments derived from the plastic zone of HVC. In such a case, there are more indigenous donor hydrogen stabilizing free radicals due to the decrease in reaction between oxygen-containing fragments and transferable hydrogen. Consequently, B_4_C may be a promising additive to improve HVC’s coking performance and coke quality.

Initially, this work aims to investigate whether adding B_4_C can improve the quality of coke obtained from HVC. Five carbonization tests were carried out in a laboratorial scale coking furnace, and the quality of resulting coke was reflected by coke reactivity towards CO_2_ (CRI) and coke strength after reaction (CSR) indexes. Secondly, one of the important tasks of this work was to acquire the mechanism of improvement of coke quality in detail. The influence of B_4_C on coking behavior during HVC carbonization was evaluated by using a thermo-gravimetric analyzer (TG). Based on the coke quality data and TG analysis, four semi-cokes with or without B_4_C were manufactured under two characteristic temperatures and were analyzed by Fourier transform infrared spectrum (FTIR) and high-resolution transmission electron microscopy (HRTEM) techniques to investigate the effects of B_4_C on chemical structure during HVC carbonization. Having a better understanding of the interactional mechanism between HVC and B_4_C contributes to increasing the proportion of low-rank coking coal (such as HVC) in coal blending, especially in the case of adding B_4_C, to produce low-cost metallurgical coke. Simultaneously, this method is easy to apply in industry.

## 2. Materials and Methods

### 2.1. Samples and Carbonization Experiments of Coke

The HVC used in the current study was collected from Kubai coal field of Xinjiang province, located in northwest China. The boron carbide (B_4_C) powder with particle diameter <58 μm had a purity of 95%. The coke carbonization experiments between HVC and B_4_C were carried out in a laboratorial scale coking furnace, and experimental schemes are shown in Table 1.

The coking furnace temperature was controlled automatically by a programmable controller and was heated by resistance wire. The density and moisture of coal blends were limited to 0.95 g/cm^−3^ and 10 wt.%, respectively. Approximately 2 kg of the coal blend (particle size of HVC less than 3 mm) was mixed evenly and placed in a coking retort with an internal diameter of 100 mm and a length of 500 mm. Next, this coking retort was put into the coking furnace after the experimental temperature reached 700 °C, combining with a heating rate 10 °C/min. After that, it was consecutively heated to 1000 °C at the rate of 5 °C/min, and the target temperature was maintained for 5 h, finally cooling the retort to room temperature in the atmosphere. The GB1997-89 standard was applied to produce coke samples whose CRI and CSR indexes were measured using GB/T4000-1996 standard. These indexes are shown as the average value of three trials in a later context.

### 2.2. TG Measurements

Generally, the carbonization process of coal is similar to its pyrolysis process under an inert gas. In order to simulate and evaluate the influence of adding B_4_C on coking behavior in the carbonization process of HVC, two groups of thermo-gravimetric analysis experiments for HVC (particle size of <74 μm in diameter) with and without 0.5 wt.% B_4_C were carried out on a NETZSCH STA 449 C analyzer (Nestal, Selbu, Germany). About 10 mg of coal blend was placed in an alumina cell and heated from ambient temperature to 1000 °C at a rate of 10 °C/min under a continuous argon atmosphere with flow rate of 50 mL/min.

### 2.3. Carbonization Experiments of Semi-Coke

To investigate the influence of B4C on the chemical structure in the carbonization process of HVC, the semi-coke carbonization experiment was carried out in an electrically heated oven using a 200 mL crucible and corresponding schemes, based on the coke quality analysis and characteristic temperatures analysis from TG, are listed in Table 1. Approximately 100 g coal blending (particle size of HVC less than 1 mm) was loaded into the crucible. The crucible was placed in the oven’s chamber filled with inert atmosphere, heated at the rate of 10 °C/min to 450 °C and 750 °C, respectively, held at the target temperature for 5 min, and then removed and cooled to room temperature under an N_2_ atmosphere.

### 2.4. Preparation of Demineralized Samples

Raw coal samples and semi-coke samples were ground and sieved to obtain particles of <74 μm in diameter. To eliminate the potential effects of minerals on the FTIR and HRTEM analyses, these samples were acid-washed using HCl and HF solution at room temperature as described in a previous study [23]. Generally, acid treatment under such conditions does not cause significant structural changes [24]. These demineralized samples were dried for 12 h in vacuum room at 60 °C and stored under nitrogen atmosphere. Ultimate analyses of these samples were determined according to GB/T 476-208 criterion and the analytical results are listed in Table 2.

### 2.5. FTIR Measurements

The FTIR spectra of the demineralized samples were recorded on a Thermo-Nicolet iS5 FTIR spectrometer (Thermo Fisher Scientific, MMAS, Waltham, MA, USA). All samples for the FTIR measurement were prepared by mixing the investigated sample with dried KBr powder, and the mixture was pressed to form a pellet under 12 MPa for 2 min. All spectra were obtained within the 400–4000 cm^−1^ wave number range at a resolution of 4 cm^−1^, and 32 scans per spectrum were performed.

### 2.6. HRTEM Measurements

The influence of adding B_4_C on lattice fringes in the carbonization process of HVC was investigated by using HRTEM. The demineralized samples were grounded in ethanol and sonicated in an ultrasonic washer for 10 min, and then sprayed on a copper microgrid as HRTEM specimens. The HRTEM images of the samples were acquired from a 200 kV transmission electron microscope (JEM-2100F, JEOL, Tokyo, Japan). Detailed procedures and conditions of HRTEM analysis were derived from a previous thesis [25].

## 3. Results and Discussion

### 3.1. Coke Quality Analysis

**Figure 1:**
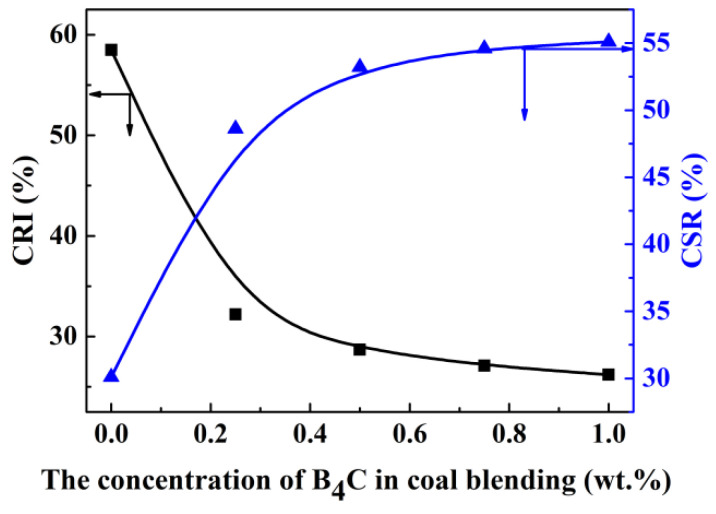
The relationship between thermal strength indexes of coke and the concentration of B_4_C in coal blending. CRI: coke reactivity towards CO_2_; CSR: coke strength after reaction.

In order to easily observe the changing trend of strength, two smooth curves in Figure 1 were automatically performed by linking data points based on the B-spline mode of software Origin 9.1. As shown in Figure 1, the CRI index of coke decreased by 26.3%, while CSR index of coke increased by 18.5% when 0.25 wt.% B_4_C was added into HVC, indicating that the coke quality is improved distinctly through the addition of B_4_C. In addition, these thermal strength indexes were further promoted when B_4_C content increased to 0.50 wt.%. However, continuing to increase the content of B_4_C to 0.75 wt.% even 1 wt.% led to little change of these indexes. These results indicate that adding 0.5 wt.% B_4_C into HVC to improve the coke quality is sufficient. Therefore, the addition amount of B_4_C was set as 0.5 wt.% in the following research on the modification mechanism.

### 3.2. Ultimate Analyses

As listed in Table 2, raw coal has a high oxygen content (16.41 wt.%, daf) and low carbon content (76.93 wt.%, daf), suggesting that raw coal is a low coalification coking coal with abundant oxygen-containing groups. In the carbonization process, the contents of hydrogen and oxygen decrease in semi-cokes while the content of carbon increases with the increase in temperature (Table 2), which is largely caused by releasing small molecular weight gases, such as CO_2_, CO, H_2_O, H_2_, etc. It is worth noting that some differences on element content and H/C ratio were observed in semi-cokes under the same temperature, indicating that B_4_C causes a change of carbonization process of HVC.

### 3.3. TG Analysis

**Figure 2:**
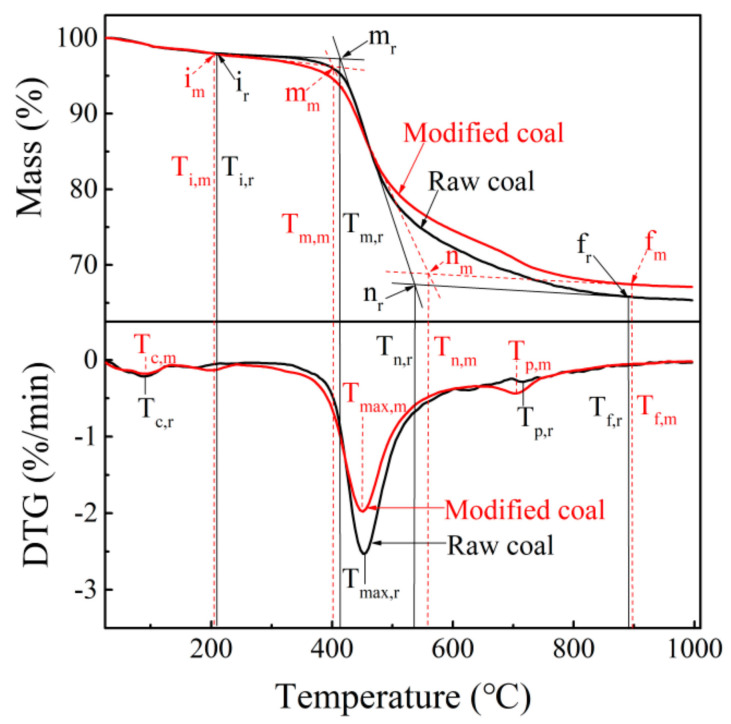
Thermo-gravimetric (TG) and DTG curves of raw coal and modified coal (addition of 0.5 wt.% B_4_C into raw coal) at a heating rate of 10 °C/min. T_*,r_: represents the characteristic temperatures of raw coal; T_*,m_: represents the characteristic temperatures of modified coal; T_c_: moisture loss peak temperature; T_i_: pyrolysis initial temperature; T_m_: initial temperature of the pyrolysis process with a fast reaction rate; T_max_: highest pyrolysis peak temperature; T_n_: finish temperature of the pyrolysis process with a fast reaction rate; T_c_: second cracking peak temperature; T_f_: pyrolysis finish temperature.

Figure 2 shows the curves of mass loss (TG) and their derivatives (DTG) for raw coal and modified coal, respectively. According to previous research [26], seven characteristic temperatures derived from the TG and DTG curves are defined and the two curves are divided into five stages as shown in Figure 2. For the drying stage (room temperature—T_i_) and slow pyrolysis stage (T_i_–T_m_; the process with a low reaction rate—Figure 2), there is little difference in both coals, indicating that the B_4_C has little effect on the dehydration process, the releasing process of gas soaked in the pores, and the decomposition process of unstable functional groups below 400 °C.

For the fast pyrolysis stage (T_m_–T_n_), the process with a fast reaction rate, a weight loss peak with a high mass loss rate (2.53 %/min) was observed in the DTG curve of raw coal, indicating that many considerable reactions occurred, and these reactions caused the formation of abundant volatile matters, free radicals, and molecular fragments in this stage. Compared with the weight loss peak, however, the corresponding peak in modified coal showed a lower mass loss rate (1.98 %/min), indicating that the reaction rate of pyrolysis reactions in the fast pyrolysis stage are slowed down by adding B_4_C. This may be because partial active oxygen obtained from thermally produced molecular fragments are combined with B_4_C to form boron oxide [21,27,28,29], which decreases the consumption of transferable hydrogen used to stabilize reactive oxygen substances so that more transferable hydrogen can be used to stabilize the free radicals. In such a case, on the one hand, more stable free radicals can be arranged in an ordered structure, resulting in the development of anisotropic mesophase structures in semi-coke [30]. On the other hand, the reaction rate of condensation and crosslinking reactions will be reduced in modified coal, which is consistent with a lower mass loss rate peak in the fast pyrolysis stage.

For the fast polycondensation stage (T_n_–T_f_), the peak at T_p,m_ is more intensive than the peak at T_p,r_, indicating that the intensity of secondary cracking reactions in modified coal is higher than that in raw coal. This is because, with the development of mesophase structures in modified coal, more macromolecular weight polymers will form and participate in secondary cracking reactions [26].

For the slow polycondensation stage (T_f_–1000 °C), there were no obvious differences in the transformation process from char to coke for both coals, but the weight of pyrolytic residues of modified coal was higher than that of raw coal after the pyrolysis finished. This is attributed not only to the residue of boron oxide, but also the lower release of small molecular weight gases in the fast pyrolysis stage.

### 3.4. FTIR Analysis

**Figure 3:**
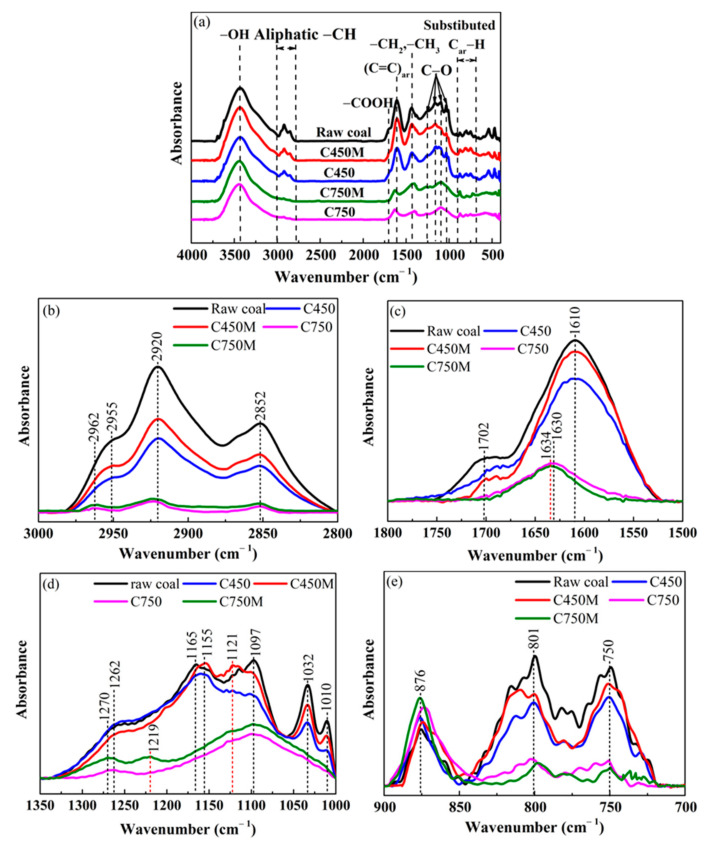
FTIR spectra of raw coal and four semi-cokes: (**a**) FTIR spectra from the 4000–400 cm^−1^ zone; (**b**) FTIR spectra after subtracting the baseline from the 3000–2800 cm^−1^ zone; (**c**) FTIR spectra after subtracting the baseline from the 1800–1500 cm^−1^ zone; (**d**) FTIR spectra after subtracting the baseline from the 1350–1000 cm^−1^ zone; (**e**) FTIR spectra after subtracting the baseline from the 900–700 cm^−1^ zone.

Figure 3a shows the FTIR spectra of raw coal and the four semi-coke samples, which exhibit similar absorption bands primarily consisting of oxygen-containing groups, aliphatic C–H groups, aromatic nucleus C=C, and substituted aromatic rings, while their intensities of absorption bands vary considerably. For further observing the changes of significant functional groups in the FTIR spectra, the baselines of zone representing aliphatic C–H groups from 3000 to 2800 cm^−1^, zones representing oxygen-containing functional groups from 1800 to 1500 cm^−1^ and 1350 to 1000 cm^−1^, and zone representing aromatic C–H groups from 900 to 700 cm^−1^ were corrected, as shown in Figure 3b–e, respectively. According to the literature [23,24,26,31,32,33], band assignments are shown in Table 3.

As shown in Figure 3a,b, the intensities of peaks at 2955 cm^−1^, 2920 cm^−1^, 2852 cm^−1^, and 1465 cm^−1^ for aliphatic C–H functional groups steadily decrease with increasing temperatures due to the thermal cleavage of aliphatic chains. Besides, these fractured aliphatic segments will participate in polycondensation reactions during aromatization to form larger condensed aromatic nucleus polymers. It is worth noting that the peak intensity at 3000–2800 cm^−1^ of C450M is greater than that of C450, indicating that C450M has a higher concentration of aliphatic C–H groups as well as a lower condensation degree of aromatic nuclei. Conversely, the content of aliphatic C–H groups of C750M are slightly lower than that of C750, suggesting that the condensation degree of aromatic nuclei in C750M increases with the addition of B_4_C.

As can be seen in Figure 3a,c,d, the intensities of peaks at 3400 cm^−1^, 1702 cm^−1^, 1262 cm^−1^, 1097 cm^−1^, 1032 cm^−1^, and 1010 cm^−1^ for oxygen-containing functional groups monotonously decrease with the increasing temperatures, even the four peaks at 1702 cm^−1^, 1165 cm^−1^, 1032 cm^−1^, and 1010 cm^−1^ disappear in semi-cokes at 750 °C. This is because these –COOH, O–H, and C–O bands are gradually broken or destroyed in the HVC carbonization process and released through small molecules gases [17,18]. It is interesting that the intensity of peak at 1610 cm^−1^ for C=C stretching band also declines with the increase in temperature. Ideally, the C=C stretching band representing the condensation degree of aromatic nuclei will increase with increasing carbonization temperature, but the presence of plenty of phenolic groups and COO– groups in low-rank HVC (C 76.93 wt.% in Table 1) is likely to increase intensity of the 1610 cm^−1^ band [34]. Therefore, the decrease in intensity of the peak at 1610 cm^−1^ is mainly due to the decomposition of oxygen-containing groups in the carbonization process of HVC.

It is worth noting that a new peak at 1121 cm^−1^ (in Figure 3d) attributed to a B–O bond is observed in C450M [29,35,36], indicating that partial active oxygen obtained from oxygen-containing fragments were consumed by reaction with B_4_C to form the boron oxide in the plastic zone of HVC. In addition, by comparing the peak strength of oxygen-containing functional groups at the same position in C450 and C450M, the strength of all oxygen-containing peaks was increased by adding B_4_C. One of hypotheses was that substitution reactions between boron compound and oxygen-containing functional groups occur, which leads to the formation of organically bound B–O groups with higher bond energies. Simultaneously, a shift of the C=C stretching band from 1630 cm^−1^ (in C750) to 1634 cm^−1^ (in C750M) and a new band at around 1219 cm^−1^ representing B–C stretching vibration are observed. Generally, an increase in wavelength of the C=C stretching band is attributed to the generation of the B–C band. Meanwhile, boron atoms should be incorporated in the sp^2^ C networks in coke structure. These two explanations are clarified in related articles [29,36]. However, compared with conditions in these articles, the experimental temperature in this work was lower. Therefore, whether the explanations introduced in the above articles are suitable to support the phenomena in this work still needs to be illustrated.

As shown in Figure 3e, these fingerprint absorption peaks at 900–700 cm^−1^ are caused by aromatic C–H out-of-plane bending vibrations. It is generally accepted that the degree of aromatic substitution and condensation of aromatic nuclei rely on the number of adjacent hydrogens per ring [24]. The intensities of peaks at 812 cm^−1^ and 750 cm^−1^ decrease gradually and the intensity of peaks at 870 cm^−1^ increases gradually with increasing temperature, indicating that the concentrations of highly-substituted aromatic rings and aromatic structures with 1–2 rings decrease in the carbonization process and the size of condensed aromatic nuclei becomes larger. It is noteworthy that the peak intensity at 876 cm^−1^ of C450M is lower than that of C450, but the peak intensity at 876 cm^−1^ of C750M is higher than that of C750 when the temperature arrives at 750 °C. Simultaneously, both peak intensities at 801 cm^−1^ and 750 cm^−1^ of C450M are higher than those of C450, but both peak intensities of C750M are lower than those of C750. These results show that the addition of B_4_C can retard condensation reactions in the plastic zone and increase the secondary cracking reactions in the fast polycondensation zone of HVC, which will lead to the formation of larger sized condensed aromatic nuclei in C750M.

### 3.5. HRTEM Analysis

**Figure 4:**
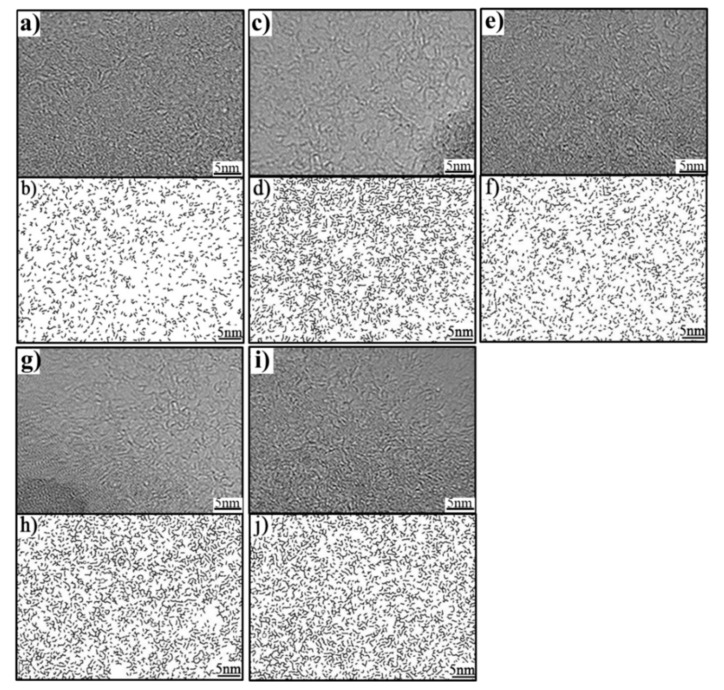
High-resolution transmission electron microscopy (HRTEM) images and the corresponding extracted lattice fringe images of coal and semi-coke samples: (**a**,**b**) raw coal; (**c**,**d**) C450; (**e**,**f**) C450M; (**g**,**h**) C750; (**i**,**j**) C750M.

The lattice fringes cannot be observed directly from HRTEM images, so these original images were organized to acquire the lattice fringe images and every step is briefly described as follows: firstly, using Fourier transformation to cope with original images obtains frequency domain images; secondly, using rounded filtering eliminates the disordered part in these images; thirdly, using inverse Fourier transformation transfers the images obtained after the second step to new images; then, using threshold segmentation to handle the new images obtains black-and-white binary images that can present microcrystalline fringe; next, initially etch, then expend, and finally skeleton process the black-and-white binary images to obtain lattice fringe images [37]. HRTEM images and the corresponding lattice fringe images of raw coal and four semi-coke samples are shown in Figure 4. According to Figure 4, these lattice fringe images show a striking difference in the shape, size, and orientation of the layers. For the raw coal, the majority of layers are small, twisted, and lack orientation; that few stacks can be observed in Figure 4b, which is consistent with its low coalification. With the elevation of carbonization temperature, aromatic layers’ stacking-number and their size increase while they become better-orientated in semi-cokes, as shown in Figure 4d,f. In addition, these changes of aromatic layers become more evident in semi-cokes at 750 °C (in Figure 4h,j). These results show that the crystallinity of semi-coke increases with the increase in carbonization temperature.

According to the literature [25,37], the number of aromatic carbon atoms can be determined by the lattice fringe length of HRTEM image; therefore, the extracted lattice fringe images were analyzed by image processing software (ImageJ V1.47) to calculate the aromatic fringe size. The relationships between lattice fringe length and aromatic sheet assignments are listed in Table 4, and the distribution frequency of lattice fringe length in the samples is also shown in Table 4 through the form of the average value based on the examination of three different regions containing 1000 aromatic fringes.

Table 4 shows a summary of the classification of the aromatic fringes by fringe lengths and their frequency of occurrence in the aromatic fringe population. For raw coal, the concentrations of benzene and parallelogram-shape aromatic structures of (<4 × 4) occupy 17.16% and 80.09%, respectively, while the ratio of parallelogram-shape aromatic structures of (>3 × 3) are only 2.75%. This result indicates that HVC contains large quantities of small size condensed aromatic structure, which are responsible for its weak thermal stability and the formation of plenty of free radicals in the plastic zone [13,16,26,38]. With increasing carbonization temperature, the concentrations of parallelogram-shape aromatic structures of (>3 × 3) increase significantly in semi-cokes, as shown in Table 4, suggesting that large quantities of condensation and repolymerization reactions occur in semi-cokes at 450 °C and 750 °C. It is noticeable that the concentration of parallelogram-shape aromatic structures of (>3 × 3) in C450M is lower than that in C450 whereas the concentration of the above structures in C750M is higher than that in C750, indicating that adding B_4_C contribute to constraining condensation reactions in the plastic zone and promoting repolymerization reactions in the fast polycondensation zone.

### 3.6. Role of the B_4_C in the Improvement of Coke Quality

B_4_C reacts with active oxygen obtained from oxygen-containing compounds in the plastic zone of HVC, which leads to reducing the reactions between active oxygen and transferable hydrogen. Therefore, more transferable hydrogens are available to stabilize free radicals, reducing the condensation and crosslinking reactions of free radicals. In this case, the anisotropic mesophase structures develop in the plastic zone of modified coal, which contributes to increasing the degree of anisotropy in coke. Simultaneously, more stable free radical fragments will be involved in the polymerization reaction under higher temperature, increasing the size of the aromatic sheet and condensed degree in semi-coke, as shown in C750M. Consequently, with further elevating temperature, the size of aromatic layer will increase in coke structures by adding B_4_C. In summary, adding B_4_C into HVC to produce coke has the result of increasing the size of the aromatic layer and anisotropic degree in the coke structure, which contributes to enhancing the coke resistance to CO_2_ reaction, ultimately resulting in the significant improvement of coke quality.

## 4. Conclusions

In this work, in addition to a new additive (B_4_C) that is primarily introduced, the modification mechanisms of coke quality were analyzed by using TG, FTIR, and HRTEM techniques. The main conclusions are summarized as follows:

(1) HVC contains large quantities of oxygen-containing functional groups, aliphatic side chains, and small molecular weight aromatic molecules, resulting in the coke derived from HVC with a high CRI index and low CSR index.
(2) B_4_C can considerably improve the quality of low-strength coke prepared from HVC. Regarding optimal thermal strength indexes, the CRI index of coke decreases by 29.8%, while the CSR index of coke enhances by 23.1% when adding 0.5 wt.% B_4_C into HVC.
(3) The reaction between B_4_C and active oxygen derived from oxygen-containing compounds during HVC carbonization leads to reduced condensation and crosslinking reactions and increased secondary cracking reactions, which result in phenomena—increasing size of the aromatic layer and anisotropic degree in the modified coke structure—that are responsible for significantly improvements of the coke quality.

## Figures and Tables

**Table 1:** Carbonization schemes for coke and semi-coke.

Samples	High Volatile Coking Coal (HVC) (wt.%)	B_4_C (wt.%)	Temperature (°C)
Scheme of Cokes			
Coke-0	100.00	0	1000
Coke-1	99.75	0.25	1000
Coke-2	99.50	0.50	1000
Coke-3	99.25	0.75	1000
Coke-4	99.00	1.00	1000
**Scheme of Semi-Cokes**			
C450	100.00	0	450
C450M	99.50	0.50	450
C750	100.00	0	750
C750M	99.50	0.50	750

**Table 2:** Elemental analyses of studied samples (wt.%, daf).

Sample	H	C	N	S	O ^a^	H/C
Raw coal	4.83	76.93	1.32	0.51	16.41	0.753
C450	4.00	81.41	1.35	0.54	12.69	0.590
C450M	4.12	80.32	1.31	0.55	13.70	0.616
C750	1.75	86.47	1.53	0.47	9.78	0.243
C750M	1.71	86.83	1.51	0.48	9.47	0.236

^a^ By difference; wt.%, daf: weight percentage of various elements on a dry and ash-free basis.

**Table 3:** Band assignments derived from FTIR spectra.

Band Position (cm^−1^)	Assignments
3415–3350	–OH stretching vibration
2975−2955	Aliphatic CH_3_ asymmetric stretching vibration
2925−2919	Aliphatic CH_2_ asymmetric stretching vibration
2855−2850	Aliphatic CH_2_ symmetric stretching vibration
1705-1695	Aromatic (carbonyl/carboxyl groups) (C=O)
1640–1605	Aromatic ring stretching C=O or C=C
1470−1450	aliphatic chains CH_3_–, CH_2_–
1274−1260	C–O stretching vibration in aryl ethers
1165−1155	C–O stretching vibration in phenols, ethers
1098−1095	C–O stretching vibration in alcohols or aromatic ring C–H bending vibration
1035−1030	Aromatic ring stretching vibration or C–O stretching vibration
1010	C–O stretching vibration
876–872	Aromatic nucleus CH, one adjacent H deformation
810–801	Aromatic nucleus CH, two adjacent H deformation
750	Aromatic nucleus CH, four adjacent H deformation

**Table 4:** Aromatic fringe assignments and distribution frequency based on the analysis of HRTEM fringe images.

Aromatic Fringe Assignments		Distribution Frequency of Aromatic Fringes (%)				
Aromatic Sheet	Grouping (Å)	Raw Coal	C450	C450M	C750	C750M
Benzene	2.5–2.9	17.16	13.57	16.05	14.20	13.06
1 × 1	3.0–5.4	52.65	41.92	50.16	40.16	39.27
2 × 2	5.5–7.4	14.66	16.32	16.01	15.45	15.84
3 × 3	7.5–11.4	12.78	15.11	13.34	14.51	13.14
4 × 4	11.5–14.4	2.14	4.44	2.08	5.49	6.39
5 × 5	14.5–17.4	0.31	2.58	0.88	3.45	3.59
6 × 6	17.5–20.4	0.20	1.62	0.88	1.57	2.37
7 × 7	20.5–24.4	0.10	1.94	0.24	1.73	1.55
8 × 8	24.5–28.4	0	0.73	0.16	1.10	1.55
>8 × 8	>28.5	0	1.78	0.16	2.35	3.35

## Data Availability

The data presented in this study are available on request from the corresponding author. The data are not publicly available due to privacy issues.
